# Commentary: Human Pathophysiological Adaptations to the Space Environment

**DOI:** 10.3389/fphys.2017.01116

**Published:** 2018-01-08

**Authors:** Joseph J. Bevelacqua, S.M.J. Mortazavi

**Affiliations:** ^1^Bevelacqua Resources, Richland, WA, United States; ^2^Diagnostic Imaging Center, Fox Chase Cancer Center, Philadelphia, PA, United States

**Keywords:** space radiation, cosmic radiation, adaptation, adaptive response, astronauts

Demontis et al. in their recently published paper entitled “Human Pathophysiological Adaptations to the Space Environment” (Demontis et al., 2017) have tried to address the main stress factors encountered in space and their impact on the human body as well as the possible lessons learned with space exploration in reference to human health on Earth. They have also tried to discuss the challenging issue of human adaptation to space environment “Humans adapt to the hostile environment of space, characterized by the absence of gravity and chronic radiation exposure, through cardiovascular adaptation and drastic changes in metabolism, respiration, body mass, bone density, and muscle integrity.” However, despite introducing the radiation risk and microgravity as the leading risks in long term manned space missions, the paper authored by Demontis et al. ignored the cardinal role of radioadaptation in deep space manned missions. Introducing the adaptive response, a phenomenon known as the increased radioresistance in cells or living organisms pre-exposed to a low adapting dose and then exposed to a high challenging dose (Scott, 2014) for effective reducing the radiation risk in long term manned space mission dates back to 2003 (Mortazavi et al., 2003a). Later, adaptive response formed the basis of many space radiation biology studies in different centers (Elmore et al., 2011; Chancellor et al., 2014; Buonanno et al., 2015; Rodman et al., 2017).

Exposure to low dose radiation can lead to activation of DNA repair and triggered apoptosis of damaged cells. To determine individual radiosensitivity, pre-exposure of lymphocytes of peripheral blood to low dose radiation can be used. This pre-exposure triggers the nonspecific defensive mechanisms which can make living organisms resistant to high dose radiation or any other detrimental agents. In this light, adaptive response can be introduced as an effective measure of individual radiosensitivity.

Although this theory is still very controversial and not generally accepted by the International community (National Research Council, 2006; ICRP, 2007), a recent NASA report entitled “Evidence Report: Risk of Radiation Carcinogenesis” (Huff et al., 2016) that is approved for public release on April 7, 2016 has cited our 2003 report as well as other reports on the importance of adaptive response studies in deep space missions “*There have been several studies performed that indicate an adaptive response to low-dose ionizing radiation can provide a level of protection against future exposures (Bhattacharjee and Ito*, *2001**; Mortazavi et al.*, *2003b**; Elmore et al.*, *2008**; Rithidech et al.*, *2012**). This may be particularly important for understanding risks in the space environment because the GCR environment is comprised predominantly of protons, and it is realistic to expect that cells will be exposed to multiple hits of protons prior to being traversed by an HZE particle*.”

Variety of effects caused by low doses of radiation such as hormesis, radioadaptive response, bystander effect (Mothersill and Seymour, 2006), should be considered when assessing radiosensitivity. Exposure to low dose radiation is associated with the effects which are linked to DNA damage and to a greater extent, to formation of reactive oxygen species (ROS) in the irradiated cells. Oxidative stress can damage the genetic material in neighboring non-irradiated cells through bystander effect. There are scientists who believe that bystander effects and genomic instability as a part of non-DNA targeted effects of ionizing radiation, can raise concerns about the risk of low dose radiation (Kadhim et al., 2013). However, the existence of an operational threshold for detrimental effects is stressed in other reports “*it is clear that adaptive responses, bystander effects and genomic instability belong to a suite of effects that predominately modulate the low dose response to radiation. These mechanisms are part of the cellular homeostatic response and, while we can detect low dose effects, there is little evidence that these translate into harm. It is likely that for many genotypes there is an operational threshold for harmful radiation damage that probably occurs at a point where the functional activity of the tissue is being compromised by the level of (protective) cell death*” (Mothersill and Seymour, 2006).

Substantial data indicates that non-targeted effects (NTE) which can be observed for low doses of high LET radiation cannot be explained by the linear dose response model used in radiation protection (Cucinotta and Cacao, 2017). It should be noted that Cucinotta et al. have recently reported that NTE, increased tumor lethality and decreased latency at high LET, as well as non-cancer mortality risks from circulatory diseases may significantly increase risk estimates to several times higher than the NASA limits (Cucinotta et al., 2017).

Mortazavi et al. have proposed that before any long-term space mission, the adaptive response of all potential crew members should be measured by routine cytogenetic tests and after *in vitro* exposure of blood lymphocytes to an adapting low dose and later to a challenging high dose and evaluation of the magnitude of the observed adaptive response, only those with high adaptive response should be chosen (Figure 1). Then, during the mission, chronic exposure to elevated levels of space radiation can considerably decrease radiation susceptibility and better protect astronauts against the unpredictable exposure to sudden and dramatic increase in flux due to solar particle events (SPEs) (Mortazavi et al., 2003a).

**Figure 1:**
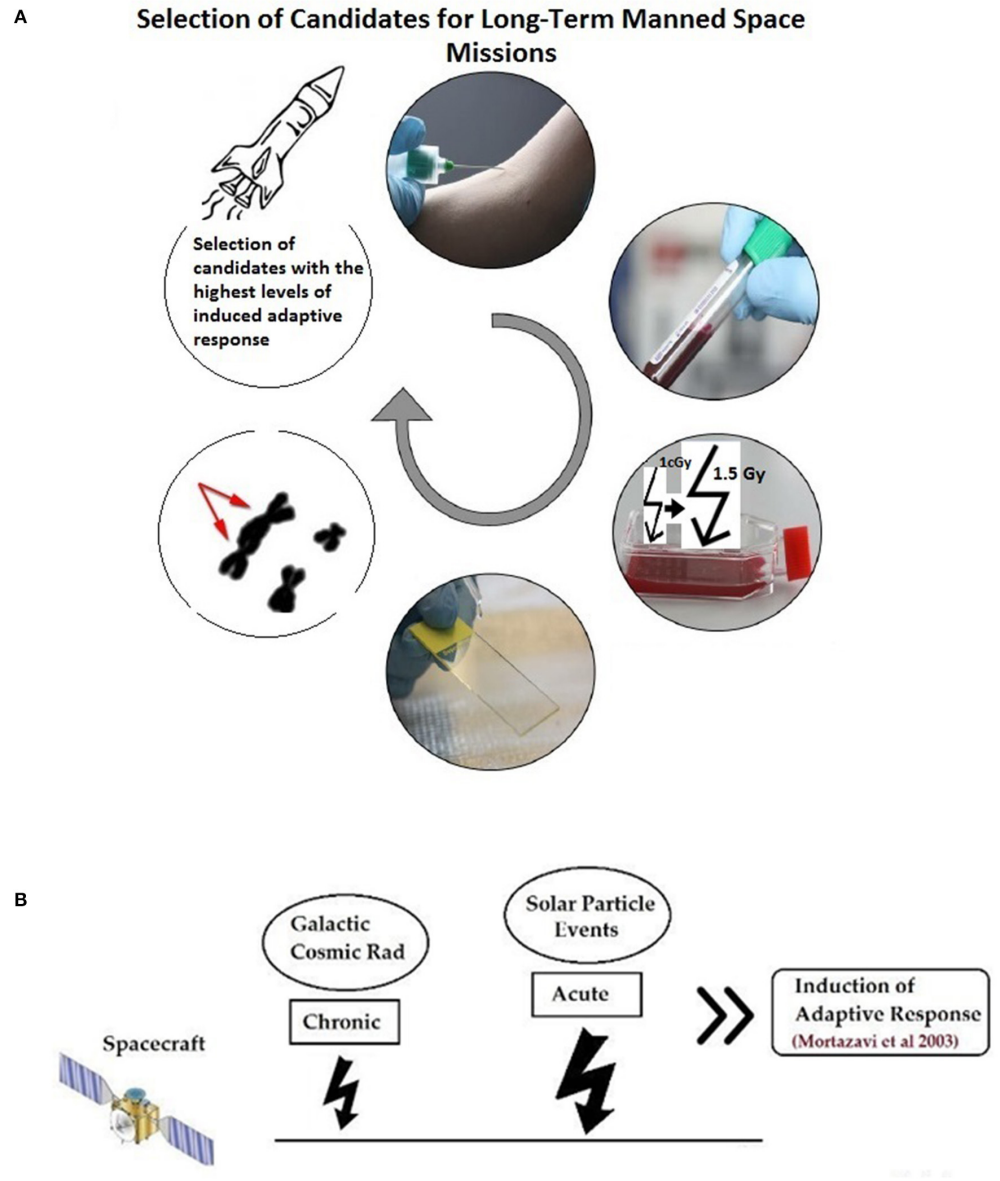
**(A)** An *in vitro* adaptive response study help choosing good candidates for deep manned space missions. **(B)** During space mission, the selected astronauts will be adapted to radiation by exposure to chronic galactic cosmic radiation (GCR) and will better tolerate sudden high doses due to solar particle events (SPE). (Figure is based on: Mortazavi et al., 2003b).

As indicated in a THESEUS report, ground-based experiments are crucial to understand the risks associated with exposure to different levels of space radiation (Worms et al., 2012). Summary of the ground-based radioadaptation tests proposed by Mortazavi et al. (Mortazavi et al., 2003a,b, 2005; Mortazavi, 2013; Mortazavi and Mozdarani, 2013) is as follows:

1. Different individuals show different levels of radioadaptation (and some show no radioadaptation and even show some kind of synergism; more adverse biological effects).
2. Candidates for deep space missions should be screened.
3. The level of the radioadaptation of each individual can be measured by some simple tests (exposing blood samples to a low adapting dose and then to a high challenging dose radiation and measuring parameters such as chromosome aberrations, DNA damage, etc.).
4. The magnitude of radioadaptation of each candidate should be determined.
5. Candidates with a high magnitude of radioadaptation will be good choices for a deep space mission.
6. During space mission, the selected astronauts will be adapted to radiation by exposure to chronic galactic cosmic radiation (GCR).
7. After adaptation, astronauts will better tolerate sudden high doses due to SPE. If a SPE occurs it can deliver potentially large doses of energetic particles even behind modest spacecraft shielding. It's worth noting that the magnitude and duration of SPE is currently unpredictable.
8. Adaptive response phenomenon can help the selected astronauts tolerate these relatively high levels of radiation.

It is worth noting that our proposed theory is in line with the experimental evidence of radioadaptation in space obtained by Durante et al. who studied chromosomal aberrations in Russian cosmonauts in multiple spaceflights. They showed an increase in chromosomal aberrations after the first mission, while for astronauts involved in multiple space flights, the yield of interchromosomal exchanges was not linked to the total duration of space mission or integral absorbed dose. They even reported that the yield of aberrations at the end of the last mission of these astronauts was in the range of background levels of aberrations measured before the first mission (Durante et al., 2003). Furthermore, the analysis of stable translocations failed to demonstrate a correlation with total days in space for astronauts who were involved in multiple missions (Durante et al., 2005). However, some studies such as the experiment conducted by George et al. (2013) failed to show any induced adaptive response. Although it can be claimed that George et al. used much larger samples and did not observe an “adaptive response,” it should be noted that the design of these studies was not appropriate, from the adaptive response point of view because the time interval between the adapting dose and challenging dose, cannot be very long. In these experiments, the authors believed that the 1st space mission could serve as the adapting dose (low dose radiation) for the next missions (challenge dose, a much higher dose of radiation). The importance of the interval between the adapting and challenge doses is previously discussed by other researchers (Elmore et al., 2011). Furthermore, none of the studies conducted so far, have used our novel protocol that is based on the screening of the candidate before a deep space mission (please refer to our 8-step protocol discussed above).

In this light, the possible role of radioadaptation in long term missions should not be ignored.

Moreover, it should be noted that we cannot equate the zero g environment and restrained earth studies “Ground-based studies represent an essential opportunity to investigate human physiology in simulated microgravity, and thus to test the effectiveness of potential countermeasures for preventing or mitigating the undesired physiological changes associated with SF, mentioned in the above paragraphs.” The authors did not pay attention to this point that Earth based studies in a gravitational field of 9.8 m/s^2^ directed toward the center of the earth are not equivalent to a zero g environment. First, earth establishes a preferential direction that affects biological processes. This force direction impacts biological processes and performing bed-rest or water immersion studies does not accurately represent the zero g environment. Second, space has no preferential direction. Biological processes influenced by gravitational force are significantly affected, but this effect is not equivalent to a constrained environment of bed-rest or water immersion.

Another issue which needs clarification in this paper is the space environment as well as the basic physics of the interaction of cosmic radiation with human body. For example, the authors believed that the penetration of space radiation is something like alpha particles (protection performed by a dead layer on our skin) or diagnostic x-rays (protection of brain by skull) “The eye is particularly susceptible to cosmic radiations, as it lacks the protections warranted to inner organs by the skin, with its layer of dead keratinocytes, or to the brain by the skull.” The physical basis of radiation protection in space is comprehensively reviewed by Durante and Cucinotta (2011). The penetrating ability of cosmic radiation is discussed in detail in current literature (Nelson, 2016) “Galactic cosmic ray ions are able to penetrate several tens of centimeters of materials such as aluminum or tissue (water) and nuclear interaction between GCR particles and target nuclei will produce lower Z secondary particles whose lower LETs confer greater range than the primary particles.” Therefore, the authors should note that the space radiation environment is complex and as discussed by Bevelacqua (Bevelacqua, 2017) some of high energy particles not only penetrate the EVA suit but also spacecraft.

## Author contributions

SM has fully reviewed and criticized the original article, drafted the commentary, reviewed, and approved the final manuscript. JB has also reviewed and criticized the original article, assisted in drafting the commentary, reviewed and approved the final manuscript.

### Conflict of interest statement

The authors declare that the research was conducted in the absence of any commercial or financial relationships that could be construed as a potential conflict of interest.
